# Animal welfare at German abattoirs: insights into the occurrence of violations against laws and regulations from official veterinarians and judicial decisions

**DOI:** 10.3389/fvets.2024.1354039

**Published:** 2024-03-06

**Authors:** Stephanie Janet Schneidewind, Susann Langforth, Diana Meemken

**Affiliations:** ^1^Working Group Meat Hygiene, School of Veterinary Medicine, Institute of Food Safety and Food Hygiene, Freie Universität Berlin, Berlin, Germany; ^2^School of Veterinary Medicine, Institute of Animal Welfare, Animal Behavior, and Laboratory Animal Science, Freie Universität Berlin, Berlin, Germany

**Keywords:** animal rights, welfare breaches, compliance and enforcement, transgressions, slaughterhouse

## Abstract

**Introduction:**

This study investigated the occurrence of various animal welfare violations at German abattoirs by analyzing the results of an anonymous online survey and relevant German court decisions.

**Methods:**

The survey targeted official veterinarians (OVs) and other individuals responsible for enforcing animal welfare laws and regulations at German abattoirs. Participants were asked to report the five most common animal welfare violations in their workplaces during the past 3 years (2019–2021), and whether 22 specific given violations had occurred or not (in the same timeframe). The court decisions were examined to determine how many abattoirs were penalized for a specific animal welfare violation and the details about the number and species of affected animals.

**Results:**

Overall, the violations examined throughout this study fell into one of the following categories: (1) use of prohibited driving aids (e.g., illicit use of electric prods, or hitting/beating animals); (2) inadequate husbandry in lairage; (3) inappropriate handling of animals with special care needs; (4) improper restraint, stunning, and bleeding of animals; and (5) unqualified task execution/inadequate administrative work (e.g., documentation of a violation). The violations analyzed in the scope of this study most frequently fell into categories 1, 2, and 4. Regarding the five violations that survey participants reported to be the most common in the years 2019–2021, 42.6% of responses (*N* = 277 reported violations) fell into category 4, followed closely by category 1 with 37.9%. Of the 22 violations surveyed in the second part of the survey, those reported by 50.0% or more participants were concentrated in categories 1, 2, and 4. Regarding the 16 relevant court rulings spanning from 2015 to 2022, the most frequently documented instances of misconduct primarily fell into category 1.

**Discussion:**

The collected data suggest that there may be need for improvements regarding compliance with animal welfare legislation, especially concerning categories 1, 2, and 4. The authors suggest OVs to consider these findings when conducting monitoring activities at abattoirs and in the training and education of abattoir personnel.

## 1 Introduction

Animal welfare during slaughter is an important ethical concern for various stakeholders, including consumers, farmers, producers, and retailers (1, 2). The subject of animal welfare during slaughter has become a point of political and social debate due to an increasing demand for ethical food production (3, 4). In Germany, an animal welfare violation at the abattoir is legally characterized as the infringement of provisions outlined in Regulation (EC) No 1099/2009 (5) and/or the German Ordinance on the Protection of Animals in Connection with Slaughter or Killing (Tierschutzschlacht-Verordnung) (6). Section 18 of the German Animal Welfare Act (Tierschutzgesetz) delineates a regulatory offense as an action perpetrated either intentionally or due to negligence (absent a justifiable cause), resulting in the unnecessary infliction of severe pain, suffering, or harm on a vertebrate (7). Section 17 of the German Animal Welfare Act defines a crime as the unjustified infliction of considerable pain or suffering upon a vertebrate, either through cruelty or by inflicting persistent or repeated severe pain or suffering. These infractions are legally and ethically unacceptable (8). Additionally, it should be considered that substandard animal welfare may have adverse effects on the quality of meat (9–11). For example, a 2014 study conducted by Dokmanović found that “rough handling” at the abattoir was correlated with increased blood lactate levels and reduced meat quality (10). In this research, “rough handling” was defined by the occurrence of at least one particular handling or behavioral parameter, or a combination thereof, including the use of a stick or electric prod, instances of pigs slipping, falling, or emitting high-pitched vocalizations.

Currently, there is no national data on the occurrence of different animal welfare violations at abattoirs. Scientific literature and official documents describe a sample of the transgressions that occurred in the past. According to Reymann in 2016 (12), animal welfare violations took place during preslaughter handling, stunning, and exsanguination in the majority of the 20 Bavarian abattoirs examined. A separate report on the inspections of Bavarian abattoirs in 2014 and 2015 found that more than 50.0% of the abattoirs inspected showed significant deficiencies, including animal welfare violations (13). However, this information may not be representative of the rest of Germany. In official printed matters, the following transgressions have been described, regarding different abattoirs across Germany: the illicit use of devices that administer electric shocks (14); an inadequate provision of feed, water, bedding and enrichment (15, 16); overcrowding and constructional deficits (16) and an insufficient restraint of animals during immobilization (allowing animals intended for slaughter to turn around, hide, resist, or even attempt to escape) (17). In 2001, Grandin described that a major cause of return to sensibility after electrical stunning in the United States was either poor bleeding or improper electric tong placement (18). In an article published in 2006, Grandin reported significant improvements in most plants between 1996 and 2003, which she attributed to the implementation of audits (19). In the same article, Grandin also described the effective stunning of bulls with a captive bolt as a problem area, but overall, facilities greatly improved animal welfare by improving stunner maintenance, installing non-slip floor gratings in stun boxes, and training staff. Nevertheless, some facilities continued to fall short during these audit assessments. In recent years, there has been a growing surge in media coverage highlighting deficiencies in the handling and slaughter of animals at abattoirs in Germany (20). Examples of violations in Germany caught on camera by animal welfare NGOs include the following: abattoir employees beating and kicking animals (especially targeting sensitive body parts) (21); the illicit use of prohibited tools to drive animals (e.g., electric prods) (22); forcefully dragging downer cattle into the abattoir instead of providing emergency slaughter as delineated in Section 8 of the German Ordinance on the Protection of Animals in Connection with Slaughter or Killing (23); and slaughtering animals despite one or more signs of consciousness after ineffective stunning (24). It is known that violations of all sorts occur, however, to the authors’ knowledge, the occurrence of different violations has not yet been investigated scientifically. Currently, there are no nationwide statistics on the frequency of various animal welfare violations in German abattoirs, given that violations recorded in individual districts are not systematically aggregated on state and federal levels by the regulatory authorities (25). To gain a better understanding of compliance and non-compliance with established regulations at German abattoirs, this study investigates the occurrence of animal welfare violations using an anonymous online-survey and available judicial decisions.

## 2 Materials and methods

The data analyzed in this research project were collected as part of a research project entitled “Development of a catalogue of measures to combat animal welfare violations at abattoirs” (German: “Erarbeitung eines Maßnahmenkatalogs zur Bekämpfung von Tierschutzverstößen im Schlachthof” –abbreviation “EMaTiSch”), which was funded by the German Veterinary Association for Animal Welfare (Tierärztliche Vereinigung für Tierschutz e.V.). The data gathered in this project were used to compile a list of measures and penalties for relevant and frequent violations of 40 provisions of the German Ordinance on the Protection of Animals in connection with Slaughter or Killing (in conjunction with the Council Regulation (EC) No 1099/2009 and the German Animal Welfare Act). For further information, see Schneidewind et al. (26). This project focused on violations at the abattoir, meaning that violations during transport to the abattoir were not addressed. The current study consisted of two major parts. Firstly, the data were obtained through an anonymous online survey and were analyzed. This survey invited official veterinarians (OVs) and other individuals responsible for upholding animal welfare laws and regulations within German abattoirs to participate, so that all professionals dealing with animal welfare at the abattoir would feel addressed. These professionals could include, for example, official veterinarians employed or authorized by competent authorities to carry out official animal welfare monitoring duties, veterinary meat inspectors, animal welfare officers, and lawyers with relevant experience.

Two separate parts of the survey provided different information on the occurrence of different animal welfare violations. In Part A of the survey, participants were asked to report the five most prevalent animal welfare violations observed in their work environments over the past 3 years (2019–2021). In Part B, they were asked to indicate whether or not 22 specific violations had occurred. Secondly, the study encompassed an examination of relevant German court decisions. All of the violations assessed in the realm of this project were divided into distinct categories: (1) use of prohibited driving aids; (2) inadequate husbandry in lairage; (3) inappropriate handling of animals with special care needs; (4) improper restraint, stunning, and bleeding of animals; and, (5) unqualified task execution/inadequate administrative work (e.g., documentation of a violation). This project received ethical approval from the Ethics Committee of the Fee University Berlin (protocol code ZEA-Nr. 2022–007; approval date: April 11th, 2022). The principles outlined in the Declaration of Helsinki were adhered to. The following sections will describe the three major components deployed to investigate the occurrence of animal welfare violations at German abattoirs.

### 2.1 An anonymous online survey among OVs

To recruit participants, an email containing the survey link was distributed to all veterinary authorities in Germany. The e-mail was sent to the reception of all veterinary offices (“Veterinärbehörden”) (*N* = 431), with the request to forward the link to colleagues working in professions that are related to monitoring animal welfare at the abattoir. The number of individual professionals who received this e-mail is unknown. This method was chosen because the contact information of OVs and other people working in a profession related to the monitoring of animal welfare in German slaughterhouses is not publicly accessible. Additionally, calls for participation were issued in two specialized journals catering to German-speaking veterinarians. Furthermore, approximately 450 attendees of an online conference in March 2022 for meat and poultry meat hygiene were informed about the survey before its launch. The e-mail invited all “Individuals with experience in monitoring animal welfare in German abattoir operations, including OVs, as well as other professionals responsible for enforcing animal welfare laws” to participate. The survey was conducted online over 2 months, running from March 1st, 2022, to April 30th, 2022. Participation in the survey was entirely voluntary, with no mandatory questions. In addition to the questions regarding the participant’s professional experience, the inquiries included questions regarding instances of infringements of Section 16 of the German Ordinance on the Protection of Animals in connection with Slaughter or Killing. The survey encompassed 22 constructed yet realistic cases of animal welfare violations, hereinafter referred to as “cases.” The majority of cases (18 out of 22) constituted regulatory offenses by Section 16 of the German Ordinance on the Protection of Animals in connection with Slaughter or Killing, as well as Regulation (EC) No 1099/2009. Some of the cases described situations that inflicted significant pain and suffering on the animals (e.g., dragging an animal that is too weak or injured to walk on their own using painful tools such as a winch or other driving aids, or slaughtering an animal without prior stunning with no respective official exemption permit), meaning that they most likely should be classified as crimes according to Section 17 of the German Animal Welfare Act.

The final set of questions and cases was organized into seven distinct question groups:

1. Questions regarding participants’ professional experiences (10 questions).

Part A (Question Nr. 2):

1. A question inquiring about the 5 most common violations encountered in the participant’s work environment (free-text entry).

Part B (Questions Nr. 3–7):

1. Cases involving violence against slaughtered animals and/or the use of prohibited driving aids (8 cases).
2. Cases of inadequate housing and husbandry of animals in lairage (3 cases).
3. Cases related to the restraining, stunning, and bleeding of animals in a manner that contravenes animal welfare standards (8 cases).
4. Cases of inappropriate handling of ill or injured animals (2 cases).
5. Cases in which employees performed tasks without the appropriate certificate of competence (1 case).

The survey was programmed using the LimeSurvey online survey tool, Version 3.28.21. Initially, participants were asked to provide information on their professional backgrounds. A complete list of the questions can be found in supplementary materials 2 and 3 in the publication by Schneidewind et al. (26) in German and English language.

The survey question in Part A [which asked participants to list the five most common animal welfare violations at the abattoir in the past 3 years (2019–2021)] was an open-ended question with no word limit. The questions in Part B inquired about the occurrence of similar cases within the participant’s work environment over the past 3 years (2019–2021). For all of the questions regarding the 22 cases in Part B, participants were presented with multiple-choice response options, along with an open-ended comment box for additional remarks. Here, participants could indicate whether the violation described had occurred in the participants’ work environment. Participants were also invited to share additional comments or insights, i.e., the specifics regarding a violation.

### 2.2 Statistical analysis of anonymous online surveys among OVs

Data analysis was conducted using IBM^®^ SPSS Statistics Version 27 (SPSS, Inc., Chicago, IL) and Microsoft Excel 2019©. This revealed which percentage of participants had encountered a specific animal welfare violation in their work environment. To provide a range within which the true population percentage can be expected to fall, 95% confidence intervals were calculated via the binomial method using the SPSS statistical software. The answers to the open-ended question in Part A were classified into one of the previously described categories: (1) use of prohibited driving aids; (2) inadequate husbandry in lairage; (3) inappropriate handling of animals with special care needs; (4) improper restraint, stunning, and bleeding of animals; and (5) unqualified task execution/inadequate administrative work (e.g., documentation). The percentage of violations falling into every category was calculated.

### 2.3 Acquisition and analysis of relevant court decisions

Judicial rulings regarding animal welfare violations that occurred during the pre-slaughter handling, stunning, and exsanguination processes at abattoirs were researched. The objective was to gather as many insights into the outcomes concerning past violations as possible, which also allowed insight into the frequencies of different violations. It is important to note that these judicial rulings do not encompass all instances of animal welfare violations. They specifically pertain to reported and severe violations that meet the criteria of a criminal act, as outlined in Section 17 of the German Welfare Act. Initially, a search within legal databases, including OpenJur (27), Juris (28), and Beck-online (29) was conducted, targeting pertinent German judicial decisions. The search exclusively focused on violations occurring within the abattoir premises. This means that transgressions that occurred during animal transportation or before arriving at the abattoir were not included. These databases yielded only a limited dataset (N = 5), since in Germany, not many animal welfare cases go to court (30). In response to the limited number of judicial rulings available, a systematic approach to sourcing additional court rulings from media reports was deployed. The search strategy involved entering specific terms into Google© (in German): “Abattoir X + Animal Welfare + Breach/Violation/Transgression” (German: “Schlachthof X + Tierschutzverstoß”). Here, “X” entailed the city where the abattoir was situated, as per a list from the year 2006, which was an online registry of approved abattoirs. This choice of 2006 as the reference year allowed for finding violations that may have occurred in abattoirs before they were eventually closed, a possibility spanning the period between 2006 and the investigation in September–October 2021. After gathering as much information as possible about breaches reported in the media (e.g., type of animal, place or name of the abattoir, file number if applicable), the press offices of 33 distinct departments of public prosecutions were contacted via email. Inquiries concerning 33 distinct animal welfare violations were made, requesting access to the corresponding judicial decisions for scientific research purposes. In Germany, the Freedom of Information Act allows persons to receive judicial decisions from courts. However, if an individual does not have the reference number (which was the case in this project for every violation apart from two), it is often not possible for the departments to identify and retrieve the specific judicial decision inquired about. As a result, there were more known cases reported in the media than court decisions obtained in this project. By the end of the project, a total of 16 German judicial decisions from the years 2015 to 2022 were obtained. The judicial decisions received were summarized and analyzed. The analysis included the following parameters:

1. How many distinct abattoirs, according to available judicial decisions, committed a specific animal welfare violation? For example, how many distinct abattoirs violated Section 5 of the German Ordinance on the Protection of Animals in Connection with Slaughter or Killing penalized?
2. How many animals and which animal species were affected by this violation according to judicial decisions?

## 3 Results

### 3.1 Results of the online survey

#### 3.1.1 Survey participants

It is unclear how many people received the e-mail, since the number of people working in a profession related to the monitoring of animal welfare in German slaughterhouses cannot be determined. Therefore, it is unknown how many persons could have participated. In total, 312 individuals started the survey. Most participants left a varying number of questions unanswered. Altogether, 204 participants ended the survey after the section regarding their profession (questions designed to screen participants). For a participant’s responses to be included in the analysis, questions corresponding to at least one case had to be answered. The answers of a varying number of participants were included in the analysis (*N* = between 66 and 108).

The first case in Part B was answered by 108 participants, and the last question was answered by 66 participants. Part A was answered by 66 participants. The variation of N was due to participants quitting the survey before the final question. The answers from participants included in the survey were from official veterinarians (OVs) (in German: “amtliche Tierärzt:innen” or “Amtstierärzt:innen”) who provided the requested information for at least one case. Other groups of professionals were not included because they did not answer the questions for at least one case (rather, they quit after the section about their professional experience). The following information describes the participant’s work experience over the past 3 years (*N* = 108). Altogether, 61.8% carried out inspections in 1–3 different abattoirs; 33.0% in 4–9 different abattoirs and 5.2% in 10 or more different abattoirs. A proportion of 68.5% of participants reported working in at least one abattoir with fewer than 20 livestock units (LU) slaughtered per week, 40.1% in at least one with 20 to 100 LU per week, and 59.8% in at least one with over 100 LU per week. The number listed in brackets describes the percentage of participants who reportedly gained experience in inspecting the slaughter of the following species in the past 3 years: pigs (87.0%), cattle (84.3%), sheep (57.4%), goats (42.6%), poultry (26.9%), horses (13.9%), and other animals (10.1%). The most common stunning method participants were experienced in inspecting was captive bolt stunning (90.5%). Other stunning methods were the following: electrical stunning (83.8%), carbon dioxide stunning (20.0%), and electrical water bath stunning for poultry (16.2%).

#### 3.1.2 Survey results

The data collected and analyzed provide indications that animal welfare-relevant deficiencies occurred throughout Germany in all stages of the slaughter process (pre-slaughter handling, stunning and bleeding) in the years 2019–2021.

##### 3.1.2.1 Survey results regarding which violations had occurred most frequently between 2019 and 2021

The first results described here are participants’ answers regarding which five violations had occurred most frequently in the past 3 years (Part B—see Question No. 7 in section 2.1). Participants were not tasked with ranking the violations from most common to least common; instead, they were asked to name the five most common violations in general. Overall, 47.0% of the survey participants (*N* = 66) reported misconduct when driving animals as one of the five most common violations in their work environment over the course of the past 3 years. The answers categorized into this group of answers were not very specific, but clearly pertained to misconduct when driving animals. Furthermore, 43.9% named the failure to properly re-stun animals that were not effectively stunned before exsanguination, and 39.4% disclosed the unauthorized use of devices that administer electric shocks to drive animals. The ranking of the frequency of the violations named can be found in Table 1. Since the question format was open-ended and not multiple choice, participants could choose to what extent they would specify the nature of the violation. Some participants named a vague violation, such as “misconduct when driving animals,” whereas others specified the illicit use of devices that administer electric shocks (e.g., too long, or in a body region that is not permitted).

**Table 1:** Percentage of survey participants naming a certain violation as one of the five most common animal welfare violations occurring in their own work environment in the past 3 years (2019–2021).

Category/Violation	n of participants (of *N* = 66 who named this violation as one of the five most common)	Percentage of *N* = 66 participants answering the question (%)	Category of violation
“Misconduct when driving animals,” not specified any further	31	47.0	1
An ineffectively stunned animal is not re-stunned before exsanguination	29	43.9	4
Illicit use of devices which administer electric shocks	26	39.4	1
Incorrect attachment of the stunning device	22	33.3	4
Inadequate supply of drinking water/feed	21	31.8	2
The time limit allowed between stunning and bleeding is exceeded	18	27.3	4
Failure to assess the effectiveness of stunning	18	27.3	4
Beating/kicking conscious animals intended for slaughter	13	19.7	1
Animals unfit for transport are delivered to the abattoir	12	18.2	1
Inappropriate handling of animals with special care needs	11	16.7	3
Use of a stunning device with visible defects/no spare equipment	9	13.6	4
Inadequate immobilization of an animal before stunning	9	13.6	4
Rough handling of animals	9	13.6	1
Overcrowding of holding pens/other spaces in the abattoir	7	10.6	1
Further preparation or scalding despite signs of consciousness	7	10.6	4
Inadequate training of employees	7	10.6	5

Altogether, 66 participants responded to this question, naming 277 violations in total. The number of the category of violation corresponds to the following: 1 = use of prohibited driving aids; 2 = inadequate husbandry in lairage; 3 = inappropriate handling of animals with special care needs; 4 = improper restraint, stunning, and bleeding of animals; and, 5 = unqualified task execution/inadequate administrative work (e.g., documentation).

Utilizing the findings presented in Table 1, the percentages of violations corresponding to the previously described categories were calculated. Table 2 illustrates the most common categories of violations according to Part A of the survey. The inclusion of this table aims to provide a clear representation of the prevalent types of violations, especially given that some responses were relatively unspecific.

**Table 2:** Distribution of violations reported among the five most common animal welfare violations reported by survey participants in their work environment over the past 3 years (2019–2021) concerning five different categories.

Category of violation	Number of violations which fell into this category (of *N* = 277)	Percentage of *N* = 277 (%)
(1) Use of prohibited driving aids	105	37.9
(2) Inadequate husbandry in lairage	23	8.3
(3) Inappropriate handling of animals with special care needs	14	5.1
(4) Improper restraint, stunning, and bleeding of animals	118	42.6
(5) Unqualified task execution/inadequate administrative work (e.g., documentation)	13	4.7

##### 3.1.2.2 Findings regarding the occurrence of 22 specific violations between 2019 to 2021

All constructed cases of the anonymous online survey have occurred in the past 3 years (2019–2021) in the working environment of participants. The most common violation was exceeding the time limit allowed between stunning and bleeding is exceeded without a certificate of exemption, which was reported to have occurred by 56.8% of the participants. The least common violation was deliberately dropping animals in transport containers, which was reported to have occurred by 6.7% of participants (for the occurrence of other violations, please see Table 3). The constructed cases were handled by a minimum of 66 persons and a maximum of 108 persons. The number of N (people who answered each question) varied because not all participants completed the survey in full, but either skipped cases or dropped out before all cases were completed. Of the 22 cases of animal welfare violations presented in the online survey, the following five violations were the most common, with the percentage of participants encountering each violation in their work environment indicated in brackets following the description: exceeding the time limit allowed between stunning and bleeding without a certificate of exemption (56.8% of *N* = 74); illicit use of instruments administering electric shocks (56.5% of *N* = 108); failure to provide animals with appropriate or sufficient feed within 6 h after arriving at the abattoir (55.0% of *N* = 80); overcrowding in holding pens (54.4% of *N* = 79); and rotating an animal’s tail by 180° for driving, in violation of Regulation (EC) No 1099/2009 (52.9% of *N* = 85). Table 3 provides an overview of the occurrence of the different cases inquired about in the online survey in Part B. All of the cases presented in the survey were included in Table 3.

**Table 3:** Frequencies of different animal welfare violations at German abattoirs in the working environments of survey participants in the past 3 years (2019–2021), ranked from most frequent to least frequent.

Rank	Percentage of participants who reported that this violation occurred in their working environment in the past (%)	Violation	Number of persons who answered this question	95% confidence interval (%)	Category of violation
1	56.8	The time limit allowed between stunning and bleeding is exceeded without a certificate of exemption, in contravention of Section 12(6)(1) of the German Ordinance on the Protection of Animals in Connection with Slaughter or Killing	74	45.4–67.4	4
2	56.5	Use of instruments which administer electric shocks in contravention of Section 5 of the German Ordinance on the Protection of Animals in Connection with Slaughter or Killing, in conjunction with Annex III(1.9) of Regulation (EC) No 1099/2009, with multiple violations occurring simultaneously (e.g., repeated, inadequately spaced out administrations of electric shocks in the hindquarters)	108	47.1–65.4	1
3	55.0	Animals not slaughtered within 6 h after arriving at the abattoir are not provided with appropriate or sufficient feed, or there are not enough troughs, or there is not a sufficient trough length per animal to ensure access to feed in contravention of Section 7(3) of the German Ordinance on the Protection of Animals in Connection with Slaughter or Killing	80	47.1–65.4	2
4	54.4	Overcrowding of holding pens, thereby not providing enough space for every animal to lie down or stand up without hindrance in contravention of Section 8(2)(1) of the German Ordinance on the Protection of Animals in Connection with Slaughter or Killing	79	43.5–64.9	2
5	52.9	Rotating an animal’s tail in contravention of Article 15(1) in conjunction with Annex III (1.8.)(e) of Regulation (EC) No 1099/2009	85	42.4–63.2	1
6	50.7	Captive bolt stunning without assessing the effectiveness of stunning in contravention of Section 4 of the German Animal Welfare Act; Section 3(1) of the German Ordinance on the Protection of Animals in Connection with Slaughter or Killing. The employee does not react to the signs of inadequate stunning	71	39.3–62.0	4
7	49.3	Sick or injured animals (which are obviously in severe pain, have large or deep wounds, are bleeding severely, or show a severely disturbed general condition) are kept in holding pens with healthy animals instead of being prioritized for immediate slaughter or euthanasia in contravention of Section 8(1)(1)(1) and (2) of the German Ordinance on the Protection of Animals in Connection with Slaughter or Killing	69	37.8–60.8	3
8	45.3	Intentionally dropping a door or a gate onto an animal, causing harm or distress	86	35.2–55.8	1
9	44.4	The attachment of the stunning device is incorrect (e.g., the bolt firing device is not positioned on the head correctly, such as not being vertical or secure, causing the bolt to be fired incorrectly or not making contact) in contravention of Article 15(1) in conjunction with Annex III(1.8)(c) of Regulation (EC) No 1099/2009	72	33.5–55.9	4
10	36.4	The required certificate of competence does not encompass the activity conducted by the employee in contravention of Article 7(2)(a) of Regulation (EC) No 1099/2009	66	25.8–48.4	5
11	32.5	Poultry arriving at the abattoir in containers are not provided with drinking water despite not being sent to slaughter within 2 h upon arrival in contravention of Section 7(2)(2) of the German Ordinance on the Protection of Animals in Connection with Slaughter or Killing	77	23.1–43.5	2
12	32.4	The bolt of the captive bolt gun has deep indentations in contravention of Article 9(1)(1) of Regulation (EC) No 1099/2009	71	22.7–43.9	4
13	28.6	Inadequate immobilization of an animal before stunning (the animal can turn around in the stun box) in contravention of Section 11 of the German Ordinance on the Protection of Animals in Connection with Slaughter or Killing	70	19.3–40.0	4
14	25.0	An animal is slaughtered without prior stunning, without a respective official exemption permit (for the purpose of Halal/Kosher slaughter) in contravention of Section 4a(2)(2) of the German Animal Welfare Act	76	16.6–35.7	4
15	24.3	Incompatible animals kept in holding pens together (e.g., dehorned and horned goats from different farms of origin), in contravention of Section 7(4) of the German Ordinance on the Protection of Animals in Connection with Slaughter or Killing, resulting in hierarchy fights	74	15.9–35.2	2
16	22.4	A pig is placed in the scalding bath without prior bleeding, in contravention of Section 12(7)(1) of the German Ordinance on the Protection of Animals in Connection with Slaughter or Killing and Article 15(1) in conjunction with Annex III (3.2)(3) of Regulation (EC) No 1099/2009	67	14.1–33.7	4
17	22.40	Dragging an animal unable to walk on its own with painful driving aids (e.g., a winch) in contravention of Section 8(1) of the German Ordinance on the Protection of Animals in Connection with Slaughter or Killing	67	14.1–33.7	3
18	21.90	Employing an electric prod on an animal that is too young according to Annex III(1.9) of Regulation (EC) No 1099/2009	96	14.8–31.1	1
19	21.80	Striking an animal with a driving stick against a sensitive body part (e.g., the eye), in contravention of Article 15(1) in conjunction with Annex III(1.8)(a) of Regulation (EC) No 1099/2009	96	13.9–30.0	1
20	11.70	Kicking an animal in the head, which violates Article 15(1) in conjunction with Annex III(1.8)(a) of Regulation (EC) No 1099/2009	94	6.6–19.7	1
21	8.60	Prohibited methods of immobilizing animals (e.g., using a bolt shot to the neck) in contravention of Article 15(3)(1) of Regulation (EC) No 1099/2009	70	3.9–17.5	4
22	6.70	Animals in transport containers are deliberately dropped in contravention of Article 15(1) in conjunction with Annex III (1.3.)(1)(a) of Regulation (EC) No 1099/2009	75	3.9–17.5	1

The number of the category of violation corresponds to the following: 1 = use of prohibited driving aids; 2 = inadequate husbandry in lairage; 3 = inappropriate handling of animals with special care needs; 4 = improper restraint, stunning, and bleeding of animals; and, 5 = unqualified task execution/inadequate administrative work (e.g., documentation).

### 3.2 Court decisions

The 16 German judicial decisions are from the years 2015 to 2022. They were comprised of seven penal orders, seven judgments, one resolution, and one dismissal notice. Notably, there were 20 negative responses to the requests for specific judicial decisions. These responses cited various reasons, including the absence of reference numbers, concerns related to data privacy, or the fact that the court cases were still pending. The violations documented in these judicial decisions encompassed a range of offenses, including the following: surpassing the permitted time between stunning and bleeding without a certificate of exemption, unauthorized use of electric shock-administering devices, physical mistreatment such as kicking or beating of animals, exsanguination of inadequately stunned animals displaying signs of consciousness (e.g., spontaneous blinking, directed eye movements, or reactions to touch), the use of painful driving aids to move weak or injured animals unable to walk on their own (e.g., the use of a winch), failure to provide animals with the requisite drinking water following pertinent regulations, and neglecting to milk lactating dairy cattle every 12 h. A summary of the court decisions obtained can be found in Table 4. Court decisions were associated with violations at 10 distinct abattoirs. The count of court decisions exceeds the number of abattoirs due to multiple verdicts for certain abattoirs. Interestingly, 13 of 16 court decisions (81.25%) were based on secret video recordings by animal welfare organizations. The remainder were based on testimonies by OVs.

**Table 4:** Occurrence of animal welfare violations in abattoirs: ranked by the number of facilities affected.

Animal welfare violation	Number of court verdicts in which this violation was penalized and year of the decision(s)	Number of abattoirs where this violation occurred (of *N* = 10 different abattoirs)	Minimum number of animals affected by this violation (not per abattoir, but overall)	Species affected with minimum number of animals affected in brackets	Reference Number(s) of court verdict(s)	Category of violation
Hitting/kicking slaughtered animals in contravention of Article 15(1) in conjunction with Annex III(1.8) of Regulation (EC) No 1099/2009	6 (4 court decisions from 2019 and 2 from 2020)	5	11	Cattle (*n* = 9), Pigs (*n* = 1), Sheep (*n* = 1)	Cs 21 Js 2416/20; Cs 21 Js 8867/18; 52 Ds 222/20; 1 Ss 93/19; 3 Cs 12 Js 7023/18; Cs 444 Js 8	1
Use of devices which administer electric shocks in contravention of Section 5(1)(1) of the German Ordinance on the Protection of Animals in Connection with Slaughter or Killing in conjunction with Annex III(1.9) of Regulation (EC) No 1099/2009	6 (3 court decisions from 2019; 2 from 2020 and 1 from 2022)	5	103	Pigs (*n* = 75), Cattle (*n* = 28)	3 Cs 12 Js 7023/18; Cs 444 Js 15,746/18; Cs 21 Js 8867/18; Cs 21 Js 8806/18; Unknown (Source: Kulmbach Local Court, penalty order from December 27th, 2021); Dismissal notice (Laatzen, 1,102 Js 76,225/18)	1
An animal that has been ineffectively stunned (e.g., one that shows signs of consciousness such as spontaneous blinking, directed eye movements or reactions to touch) is not re- stunned before exsanguination	3 (1 court decision from 2018 and 2 from 2020)	3	49	Cattle (*n* = 43), Pigs (*n* = 6)	9 Ns – 9,634 Js 23,170/13; 2 Ss 194/20; 590 Js 10,044/16	4
Dragging an animal unable to walk on its own with painful driving aids (e.g., a winch) in contravention of Section 8(1) of the German Ordinance on the Protection of Animals in Connection with Slaughter or Killing	4 (3 from 2020, 1 from 2021)	3	12	Cattle	23 Cs (1,102 Js 47,536/20) 538/20; 23 Ds (1,102 Js 23,602/20) 282/20; Cs 444 Js 15,746/18; Cs 444 Js 17,063/18;	3
Poultry arriving at the abattoir in containers are not provided with drinking water despite not being sent to slaughter within 2 h upon arrival in contravention of Section 7(2)(2) of the German Ordinance on the Protection of Animals in Connection with Slaughter or Killing	1 (2015)	1	752	Turkeys	11 A 3678/14	2
Failure to milk lactating dairy cattle at least every 12 h in contravention of Annex III(1.5)(2)(a) of Regulation (EC) No 1099/2009	1 (2018)	1	136	Cattle	590 Js 10,044/16	4
The time limit allowed between stunning and bleeding is exceeded without a certificate of exemption (in accordance with Section 13(2) of the German Ordinance on the Protection of Animals during Slaughter or Killing)	1 (2018)	1	130	Cattle	590 Js 10,044/16	4

## 4 Discussion

The objective of this study was to assess the occurrence of different transgressions related to animal welfare laws and regulations in German abattoirs. This topic warrants investigation, so that strategies to optimize animal welfare at abattoirs can be developed: e.g., improving the education of abattoir personnel (31) and advising official veterinarians (OVs) on which steps in the process of slaughtering animals require more frequent and extensive monitoring. The findings from the online survey and analysis of court decisions showed that animal welfare violations at German abattoirs most frequently fall into categories 1 (use of prohibited driving aids), 2 (inadequate husbandry in lairage), and 4 (improper restraint, stunning, and bleeding of animals). However, it is crucial to contextualize the obtained results to derive meaningful insights. For instance, a single violation at an abattoir might have far-reaching consequences for numerous animals, as illustrated in Table 4. Consequently, the interpretation of violation frequency remains contingent on whether one examines the number of abattoirs where such breaches occurred or the magnitude of animals impacted. Moreover, the information sources play a pivotal role in determining frequencies. The dual components of the online survey yielded a marginally different ranking compared to the outcomes derived from the analysis of court verdicts. This variance is expected, considering that not all violations result in court trials. Typically, it is the more severe cases that find their way into court, while less severe transgressions are addressed by alternative authorities. Additionally, not even all severe cases go to court (32). Recognizing this dichotomy is essential for a comprehensive understanding of the reported frequencies. The findings of this study reveal a notable frequency of animal welfare violations within German abattoirs. Among the 22 specific violations examined, five were reported to occur in over 50.0% of the surveyed participants’ working environments. If comparable findings can be gained in larger, more standardized studies, it would indicate that there is a need for significant enhancements in the enforcement of animal welfare laws and regulations. The reasons for this occurrence of animal welfare violations may lie in deficits regarding law enforcement (8). In the context of farm animals, infringements against the German Animal Welfare Act are often dismissed (33). Moreover, Thilo (34) found no significant relationship between the severity of animal welfare violations and the outcome of the proceedings. The data gathered on animal welfare violations according to a voluntary survey and obtainable judicial decisions suggests that animal welfare conditions at abattoirs may need improvement. Understanding which violations are especially prevalent can inform strategies for improvement and provide incentives to improve practices. According to Fötschl (35), it is particularly important for OVs to repeatedly point out misconduct to slaughterhouse personnel and impose the necessary measures. The findings of this study could support such efforts. Improving animal welfare at the abattoir is important from an ethical standpoint, given the occurrence of unwarranted pain and suffering. In this context, the results of this study may serve as an incentive for abattoir operators to assume greater responsibility in addressing violations and improving the monitoring of animal handling practices. To the authors’ knowledge, this project presents the first study of its kind. The results obtained could be explained by the statement made in an article by Scheibl (36), which describes that in many cases, the cause of an animal welfare violation is that the involved parties lack sufficient knowledge regarding the handling of slaughter animals. The author describes that the affected group includes individuals ranging from ordinary workers to managerial personnel, and that the primary focus for improving the situation should be on providing the necessary knowledge in the form of continuing education for both experienced butchers already in the workforce and those undergoing initial training (37). Furthermore, according to Braunmiller (38), the fines imposed to sanction animal welfare violations do not have a deterrent effect. To some extent, this could possibly account for the findings of this study. According to the study published by Reymann in 2016, animal welfare violations occurred during preslaughter handling, stunning, and exsanguination in the majority of the 20 largest Bavarian abattoirs audited (12). The study reported on violations occurring during unloading in the holding pen, driving animals, stunning, bleeding and documentation/management. The findings of Reymann’s study align with the outcomes of the present study, as both studies reveal that all aspects related to animal handling and care can be improved. Instances of animal welfare violations often stem from a variety of factors, including negligence of abattoir employees or operators, or specific challenges such as structural deficiencies, as reported in the works of Fötschl (35), Scheibl (36, 37), and Hahn and Kari (20). Furthermore, these transgressions can be linked to additional factors, such as time constraints, high slaughter rates, inadequate infrastructure, or economic interests (39). The most evident factor to tackle is a lack of education and training for the tasks personnel are hired for. According to Nicolaisen et al. in 2023 (40), the training program they developed holds the promising potential to enhance animal welfare practices, alleviate stress levels for both workers and animals, and create a more favorable overall work environment (41).

A limitation of data collected through a voluntary and anonymous survey is that the information reported cannot be verified. This is a common challenge associated with survey research (42). However, by using this methodology, participants were allowed to disclose which violations occurred in their working environments in the past 3 years without concerns about potential identification and respective consequences about data protection. This aspect is particularly significant given the subject matter, as there has been media coverage highlighting concerns about OVs inadequately responding to animal welfare violations (43). However, due to the anonymous nature of the survey, the findings may not generalize to the entire country. It cannot be ruled out that violations that occurred at one abattoir were reported more than once. For instance, it is conceivable that both the animal welfare officer and the responsible OVs working at the same abattoir may have reported on the same cases. Moreover, given the uncertainty surrounding the number of individuals who received or could have received the email, the representativeness of the obtained results cannot be determined. It cannot be excluded that violations were over- or under-reported. Additionally, the number of incidents of animal welfare violations is likely much higher when OVs are not present for monitoring. This could mean that the incidents reported in the online-survey is most likely lower than the actual number of animal welfare violations which occurred in the timeframe studied. Professionals who encountered a higher number of animal welfare violations in their work environments may have been more inclined to participate in this survey under such circumstances. Consequently, individuals with little or no such experiences might have been less motivated to engage in the survey. Moreover, one would expect more compliance with animal welfare laws and regulations when OVs are present. To this end, the fact that 81.25% of the court decisions were based on secret video recordings heavily suggests that the number of animal welfare violations is under-reported. Additionally, when OVs are monitoring, there may be differences in inter-observer reliability, meaning that some of the OVs could lack adequate training to identify breaches of animal welfare laws and regulations (e.g., not identifying signs of recovery of consciousness). Furthermore, the interpretation of animal welfare laws and regulations can be rather subjective (44). Also, some OVs see breaches of animal welfare violations, but do not document them out of fear of retaliation (30). As for the court decisions, the limitations regarding generalizability also apply, especially due to the low number of court decisions available. It is to be expected that with 317 abattoirs across Germany (not including poultry slaughter) (source: BMEL), there will be significantly more judicial violations of animal welfare legislation. The fact that there are only 16 court decisions that could be an indication that there is a low number of animal welfare violations in Germany which get reported as crimes, but could also mean that law enforcement is inadequate. Therefore, the representativeness can only be speculated. These results serve as a first indication that compliance with animal welfare laws and regulations may be inadequate. However, it is essential to validate these findings through future studies. Regarding the collected judicial decisions, there were constraints which restricted the number of violations that could be examined within the scope of this study. While there are additional court decisions beyond those included in this research, their inclusion was hindered by the factors outlined in the results section (3.2). However, it is often the case that severe animal welfare violations are not proceeded in court, due to reasons such as inadequate documentation and procedural errors (33), meaning that the number of actual court cases concerning animal welfare violations at the abattoir may be relatively low. A very interesting and relevant question in field of research regarding animal welfare violations at abattoirs is the role of abattoir size on the occurrence of violations. However, investigating this was not feasible with the data obtained in this survey. Most participants had experience monitoring animal welfare in abattoirs of different sizes and were not asked to indicate whether a violation had a occurred at a smaller or larger abattoir. This could be investigated in future studies. Future studies could also assess violation frequencies over time to identify whether there are any trends (e.g., differences after implementing mandatory video surveillance or implementing artificial intelligence to identify non-compliance). Additionally, similar studies should be conducted in other countries. Subsequently, a comparative analysis could be conducted to identify which countries practice animal handling the best and how these findings can be considered in German policymaking. Afterwards, the impact of different interventions (e.g., training programs for abattoir staff) could be evaluated. Future studies could also develop metrics to help OVs assess animal welfare violations. Also, a similar study could be conducted to investigate animal welfare during transport to abattoirs, seeing as this is also known to be a critical part of the process in which various animal welfare violations occur (45).

The most important practical implication this study aims to achieve is an improvement of industry practices. Apart from more extensive training on animal welfare standards and best practices, the number of internal and external audits may increase to ensure that suppliers are sourcing meat from abattoirs that comply with animal welfare standards. In the future, should the findings be confirmed by additional studies, they could serve as the foundation for establishing certifications and labels for meat products that adhere to elevated animal welfare standards at abattoirs.

## 5 Conclusion

The objective of this study was to investigate the occurrence of various animal welfare violations occurring at German abattoirs, encompassing violations in five different categories (use of prohibited driving aids; inadequate husbandry in lairage; inappropriate handling of animals with special care needs; improper restraint, stunning, and bleeding of animals; and unqualified task execution/inadequate administrative work). It employed two major parts: an analysis of data collected in an online survey among individuals responsible for enforcing animal welfare regulations at abattoirs, and an analysis of available relevant court decisions. Violations were reported to occur most frequently in the following categories: use of prohibited driving aids; inadequate husbandry in lairage; and improper restraint, stunning, and bleeding of animals. Five specific infractions pertaining to these categories were reported to have taken place in the working environments of over 50.0% of the surveyed participants between 2019 and 2021. The findings of this research project suggest that there may be a need to improve the implementation of animal welfare laws and regulations, but this needs to be validated in future studies. The results are a first step toward improving monitoring activities and shaping training programs for abattoir personnel.

## Data availability statement

The raw data supporting the conclusions of this article will be made available by the authors, without undue reservation.

## Ethics statement

This project received an ethical approval from the Ethics Committee of Freie Universität Berlin (protocol code ZEA-Nr. 2022–007; approval date: April 11th, 2022). The studies were conducted in accordance with the local legislation and institutional requirements. The participants provided their written informed consent to participate in this study.

## Author contributions

SS: Conceptualization, Data curation, Formal analysis, Investigation, Methodology, Software, Visualization, Writing – original draft, Writing – review & editing. SL: Conceptualization, Data curation, Funding acquisition, Project administration, Resources, Supervision, Validation, Writing – review & editing. DM: Conceptualization, Data curation, Funding acquisition, Project administration, Resources, Software, Supervision, Writing – review & editing.
